# Changes in Intestinal Flora and Serum Metabolites Pre- and Post-Antitumor Drug Therapy in Patients with Non-Small Cell Lung Cancer

**DOI:** 10.3390/jcm13020529

**Published:** 2024-01-17

**Authors:** Zhenyu Tian, Yan’e Liu, Dan Zhu, Baoshan Cao, Ming Cui

**Affiliations:** 1 Department of Cardiology, Institute of Vascular Medicine, Peking University Third Hospital, Beijing 100191, China; tzy003@126.com (Z.T.); andrea_zhu@163.com (D.Z.); 2 Department of Cancer Chemotherapy and Radiation, Peking University Third Hospital, Beijing 100191, China; liuyane0722@163.com

**Keywords:** gut microbiota, non-small cell lung cancer, metabolism, drug therapy, metabolomics

## Abstract

Objective: this study aimed to identify the relationships between gut microbiota, metabolism, and non-small cell lung cancer (NSCLC) treatment outcomes, which are presently unclear. Methods: in this single-center prospective cohort study, we investigated changes in the gut microbiota and serum metabolite profile in 60 patients with NSCLC after four cycles of anticancer therapy. Results: The microbial landscape of the gut exhibited a surge in Proteobacteria and Verrucomicrobiota populations, alongside a decline in Firmicutes, Actinobacteriota, and Bacteroidota. Furthermore, a significant shift in the prevalence of certain bacterial genera was noted, with an increase in Escherichia/Shigella and Klebsiella, contrasted by a reduction in Bifidobacterium. Metabolomic analysis uncovered significant changes in lipid abundances, with certain metabolic pathways markedly altered post-treatment. Correlation assessments identified strong links between certain gut microbial genera and serum metabolite concentrations. Despite these findings, a subgroup analysis delineating patient responses to therapy revealed no significant shifts in the gut microbiome’s composition after four cycles of treatment. Conclusions: This study emphasizes the critical role of gut microbiota changes in NSCLC patients during anticancer treatment. These insights pave the way for managing treatment complications and inform future research to improve patient care by understanding and addressing these microbiota changes.

## 1. Introduction

Lung cancer is the leading cause of cancer-related mortality worldwide, with an estimated 1.8 million deaths annually [1]. Non-small cell lung cancer (NSCLC) accounts for approximately 85% of all lung cancer cases [2]. NSCLC has two major histologic subtypes: adenocarcinoma and squamous cell carcinoma. Several treatment modalities are available for NSCLC, including surgery, radiation therapy, anticancer therapy, targeted therapy, and immunotherapy. The choice of therapy depends on the disease stage, molecular profile of the tumor, and overall condition of the patient.

NSCLC predominantly arises in the epithelial cells of the lung [3]. Numerous genetic and epigenetic alterations are involved in the development and progression of NSCLC, comprising mutations in key genes such as epidermal growth factor receptor (EGFR), ALK tyrosine kinase receptor (ALK), and Kirsten rat sarcoma viral oncogene homolog (KRAS) [4]. These molecular changes facilitate uncontrolled cellular proliferation, leading to the formation of a malignant tumor. Risk factors for NSCLC include cigarette smoking, exposure to environmental carcinogens, and a family history of lung cancer [5].

NSCLC remains a significant public health concern, and various treatment strategies have emerged over the years. Advances in anticancer therapy, targeted therapy, and immunotherapy have revolutionized the management of NSCLC, leading to improved patient outcomes. However, despite its therapeutic benefits, anticancer therapy is associated with numerous short-term and long-term side effects. It may cause a reduction in hematopoiesis, leading to anemia, neutropenia, and thrombocytopenia [6]. Some chemotherapeutic drugs, such as anthracyclines and fluoropyrimidines, are associated with long-term cardiovascular side effects, including cardiomyopathy and heart failure [7]. Anticancer therapy-induced lung diseases can manifest as pulmonary fibrosis or pneumonitis, leading to long-lasting dyspnea and decreased lung function [8]. In rare cases, therapy-related secondary malignancies have been associated with chemotherapeutic treatment for lung cancer [9].

The adverse effects of anticancer therapy on patients with NSCLC considerably influence the quality of life and overall health of patients. These considerations highlight the importance of ongoing research to develop novel therapies with improved safety profiles and better-tolerated side effect management strategies.

Recent findings suggest that the gut microbiota plays crucial roles in the efficacy and toxicity of chemotherapeutic agents. The gut microbiome can modulate the absorption, distribution, metabolism, and excretion of drugs, thereby influencing their pharmacokinetics and pharmacodynamics [10]. The gut microbiota can also affect the immune system of the host, thereby modulating responses to anticancer therapy [11]. Preclinical studies in mice showed that the gut microbiota enhanced the efficacy of PD-1-based immune checkpoint inhibitors by promoting T cell infiltration into the tumor microenvironment [12]. Currently, several studies have explored the changes in the microbial composition of NSCLC patients. Previous research has demonstrated that abnormalities in the gut microbiome and metabolomics are closely associated with the onset and progression of early-stage non-small cell lung cancer (NSCLC). Multi-omics analyses have further identified potential associations between certain gut microbial communities and the serum phospholipids and fatty acids in patients with early-stage NSCLC, providing a foundation for further research into the pathogenesis and treatment of NSCLC. However, most of them focus on the comparison between patients diagnosed with NSCLC and healthy individuals or on cross-sectional studies examining a specific time point after treatment [13,14,15]. 

In this study, we aimed to determine the relationship between the gut microbiota and metabolism of NSCLC patients during treatment, striving to offer new insights into the prevention and management of drug-related complications in NSCLC from the perspective of the gut microbiome.

## 2. Materials and Methods

### 2.1. Ethics

The Ethics Committee of Peking University Third Hospital approved the study design (M2021598). All patients involved in this study provided written informed consent. In addition, the clinical study was registered with the American Registry of Clinical Trials (NCT05728788). This study was conducted as a subproject of the registered research.

### 2.2. Study Design and Patient Enrollment

This was a single-center prospective cohort study. The follow-up period was 3 months, and the preliminary results were evaluated in the 3rd month. No similar studies were previously reported that could serve as a reference. In total, we recruited 60 patients during visits who were diagnosed with NSCLC and scheduled for treatment with antineoplastic drug treatment in the Department of Cancer Chemotherapy and Radiology of Peking University Third Hospital from 1 July 2022 to 1 May 2023. The inclusion and exclusion criteria are presented in Table 1. Data for all patients were collected before starting the treatment, and rigorous follow-up analyses were performed after they received four cycles of antineoplastic therapy. Stool samples were collected, and 30 patients were randomly selected for serum sample analysis of intestinal microbial metabolites.

The present study attempted to evaluate the impact of anticancer therapy drugs on the gut microbiota of patients with NSCLC before and after the commencement of therapy. In light of ethical considerations, withholding chemotherapeutic treatment from patients with NSCLC to generate a traditional control group is not permissible as anticancer therapy remains a fundamental treatment modality for these patients [16,17]. Thus, this investigation employed a self-controlled design, where baseline measurements of each patient served as their own control. This design compensates for inter-individual variability and aligns with ethical standards for clinical research. From a statistical perspective, our study leverages repeated measures analysis, thereby considering the within-subject design [18,19]. Depending on the complexity of the data, further analyses could be conducted using advanced models such as mixed-effects models or time series analyses [20]. Therefore, the design of this study balances ethical standards with rigorous scientific standards, offering crucial insights into the interaction between anticancer therapy and the gut microbiota in patients with NSCLC.

### 2.3. Clinical Parameters

Blood samples were collected to determine complete blood counts (WBC, Hb, and PLT levels), identify the markers of myocardial injury (NT-proBNP, creatine kinase isoenzymes MB (CK-MB), and cTnT), assess liver function (alanine aminotransferase (ALT), aspartate aminotransferase (AST), albumin (ALB), total bilirubin (TBIL), direct bilirubin (DBIL), γ-glutamyl transaminase (GGT), and alkaline phosphatase (ALP) levels), and evaluate renal function (blood urea nitrogen (BUN) and creatinine (Cr) levels). Echocardiography was performed for determining the cardiovascular side effects. Biochemical and hematological analyses were conducted using the fully automated hematology analyzer Sysmex XE-2100 and the fully automated biochemistry analyzer Beckman Coulter AU2700. Fecal samples were used for 16S rRNA gene sequencing, and additional serum samples were used for intestinal flora metabolite analysis.

### 2.4. Sample Collection

The patients were instructed to fast for at least 8 h before collecting their fecal and serum samples early in the morning. The fecal samples were divided into four equal portions (approximately 200 mg/portion) and placed in sterile cryovials. The samples were snap-frozen in liquid nitrogen for 30 min and then immediately stored in a sterile −80 °C freezer for subsequent analysis. After obtaining the blood samples, they were gently mixed approximately 10 times, transferred into sterile cryovials, and immediately transported to the laboratory. Serum samples were obtained by centrifuging the blood samples at 1800× *g* for 10 min at 4 °C and transferring the supernatants to 1.5 mL centrifuge tubes. Each supernatant was then centrifuged again at 13,000× *g* for 2 min at 4 °C, and the resulting supernatants were transferred to fresh cryovials. The samples were snap-frozen in liquid nitrogen for 4 h and stored at −80 °C for further analysis.

### 2.5. DNA Extraction

DNA was extracted from the samples using cetyl trimethyl ammonium bromide (CTAB) according to the manufacturer’s instructions. The reagent, which was designed to uncover DNA from trace amounts of a sample, has been shown to be effective for the preparation of DNA of most bacteria. Nuclear-free water was used for a blank. The total DNA was eluted in 50 μL of Elution buffer and stored at −80 °C until measurement in the PCR by LC-Bio Technology Co., Ltd., Hangzhou, Zhejiang Province, China [21].

### 2.6. PCR Amplification and 16S rDNA Sequencing

The 5′ ends of the primers were tagged with specific barcodes tailored for each sample, complemented by sequencing universal primers. PCR amplification was conducted using a 25 μL reaction mixture, comprising 25 ng of template DNA, 12.5 μL PCR Premix, and 2.5 μL of each primer, and the volume was adjusted with PCR-grade water. The amplification conditions for the prokaryotic 16S fragments were as follows: an initial denaturation at 98 °C for 30 s; followed by 32 cycles of denaturation at 98 °C for 10 s, annealing at 54 °C for 30 s, and extension at 72 °C for 45 s; concluding with a final extension at 72 °C for 10 min. The success of the PCR amplification was verified through 2% agarose gel electrophoresis. Throughout the DNA extraction procedure, ultrapure water was utilized in place of the sample solution to serve as a negative control, ensuring no false-positive PCR outcomes. The resultant PCR products were purified using AMPure XT beads (Beckman Coulter Genomics, Danvers, MA, USA) and their concentrations were determined with Qubit (Invitrogen, Waltham, MA, USA). Amplicon pools were readied for sequencing, and both the size and quantity of the amplicon library were assessed using an Agilent 2100 Bioanalyzer (Agilent, Santa Clara, CA, USA) and the Library Quantification Kit for Illumina (Kapa Biosciences, Woburn, MA, USA), respectively. Sequencing was performed on the NovaSeq PE250 platform [22].

### 2.7. Microbiome Data Analysis

Paired-end reads were matched to their corresponding samples based on their unique barcodes and were later truncated by removing the barcode and primer sequences. The paired-end reads were combined using FLASH software (version 1.2.8) [23]. Subsequently, raw reads were subjected to quality filtering to obtain high-quality clean reads using fqtrim software (version 0.94). Vsearch software (version 2.3.4) was employed to filter out chimeric sequences. After dereplication, a representative sequence with single-base accuracy, that is, the ASV feature table and sequence, was obtained using DADA2 software (version 2019.7). QIIME2 software (version 2019.7) was utilized to calculate alpha- and beta-diversity values after normalization to random sequences. Graphs were generated using R software (version 4.3.1). To annotate the characteristic sequences of each representative sequence, each sequence was aligned using BLAST with the SILVA database. Linear discriminant analysis effect size (LEfSe) analysis and Wilcoxon rank-sum tests were used to identify differentially abundant genera between different groups of samples [24]. The main purpose of LEfSe analysis was to identify species (biomarkers) with significant differences in abundance between different groups. The analysis steps primarily involved the Kruskal–Wallis rank-sum test to detect all feature species and identify significant abundance differences of species between different groups. The Wilcoxon rank-sum test was utilized to identify species with significant abundance differences (identified in the previous step) that tend to converge at the same classification level. Linear discriminant analysis was used to select a final set of species as biomarkers. Various diagrams were created using R and GraphPad Prism (version 9.4.0; GraphPad Software, San Diego, CA, USA) software [25].

### 2.8. Analysis of Serum Samples

Metabolites were extracted from the serum samples using 80% methanol. Specifically, 400 µL of pre-chilled 80% methanol was added to 100 µL of the sample and vortexed for 1 min. The mixture was then incubated for 5 min at 25 °C, followed by overnight storage at −20 °C. After centrifuging the samples at 4000× *g* for 20 min, the supernatants were transferred to individual wells in a 96-well plate. Quality control samples were prepared by pooling 10 μL of each extract. The metabolites were stored at −80 °C before conducting liquid chromatography–mass spectrometry (LC-MS) analysis [26].

### 2.9. Non-Targeted Metabolomics Analysis

Ultrahigh performance LC-MS/MS analyses were conducted using the Vanquish UHPLC system (Thermo Fisher Scientific, Karlsruhe, Germany) coupled with an Orbitrap Q ExactiveTM HF-X mass spectrometer (Thermo Fisher Scientific, Germany). Each sample was injected into a Hypersil Gold column (100 × 2.1 mm, 1.9 µm) using a 12 min linear gradient at a flow rate of 0.2 mL/min. When running the samples in the positive-polarity mode, the eluent comprised 0.1% formic acid–water (A) and methanol (B). However, in the negative-polarity mode, 5 mM ammonium acetate with a pH of 9.0 (A) and methanol (B) were used as eluents. The Orbitrap Q ExactiveTM HF-X mass spectrometer was operated in the positive/negative mode with a spray voltage of 3.5 kV, capillary temperature of 320 °C, sheath gas flow rate of 35 pounds per square inch, auxiliary gas flow rate of 10 L/min, and auxiliary gas heater temperature of 350 °C [27]. To intuitively elucidate the relationships between samples and metabolite-abundance differences between the two groups, we performed KEGG pathway enrichment analysis on the differentially abundant metabolites.

### 2.10. Statistical Analysis

Patient characteristics were expressed as the mean ± standard deviation (SD), and differences between groups were compared using a chi-square test or a paired-samples *t*-test. The Wilcoxon rank-sum test (for two groups), the Kruskal–Wallis test (for more than two groups), and the mixed-effects model were used to compare differences among multiple groups. Student’s *t*-test and fold-change analysis were used to compare metabolites between different groups. Spearman rank correlation analysis was performed to evaluate relationships between microorganisms and metabolites. A *p*-value of <0.05 was considered to reflect a statistically significant difference. The data were analyzed using R software (version 4.3.1), IBM SPSS Statistics (version 27.0, IBM Corp., Armonk, NY, USA), and GraphPad Prism. We used partial least squares discriminant analysis (PLS-DA) as a supervised multivariate statistical analysis method to maximize the screening for differential metabolites across groups. 

## 3. Results

### 3.1. General Clinical Findings

In total, 60 patients who were diagnosed with NSCLC and scheduled to receive regular anticancer therapy were enrolled. Among these patients, 34, 25, and 1 were diagnosed with squamous carcinoma, adenocarcinoma, and large cell carcinoma, respectively. All patients received standardized antineoplastic drug therapy; the specific treatment plan is shown in Table 2. Clinical indicators related to cardiac injury, liver function, and renal function were statistically analyzed (Table 3).

### 3.2. Gut Microbial Profile

To investigate whether the changes in the gut microbiota were associated with early myocardial injury related to tumor treatment, we sequenced 16S rRNA genes in fecal microbiota samples from 60 patients before and after anticancer therapy. It must be noted that no patients enrolled in this study had a definitive diagnosis of cardiovascular disease.

**Figure 1:**
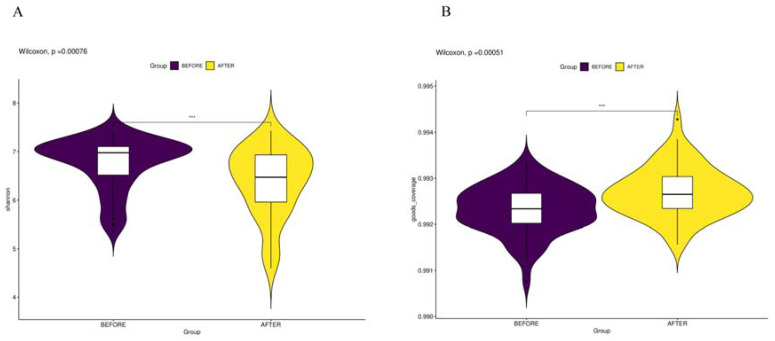
(**A**) “Shannon Index and Biodiversity”: represents information entropy with higher values signifying increased community diversity. (**B**) “Goods Coverage of Microbial Species”: indicates the comprehensiveness of microbial sequencing, with higher values suggesting more complete species representation in the sample. *** *p* < 0.001.

**Figure 2:**
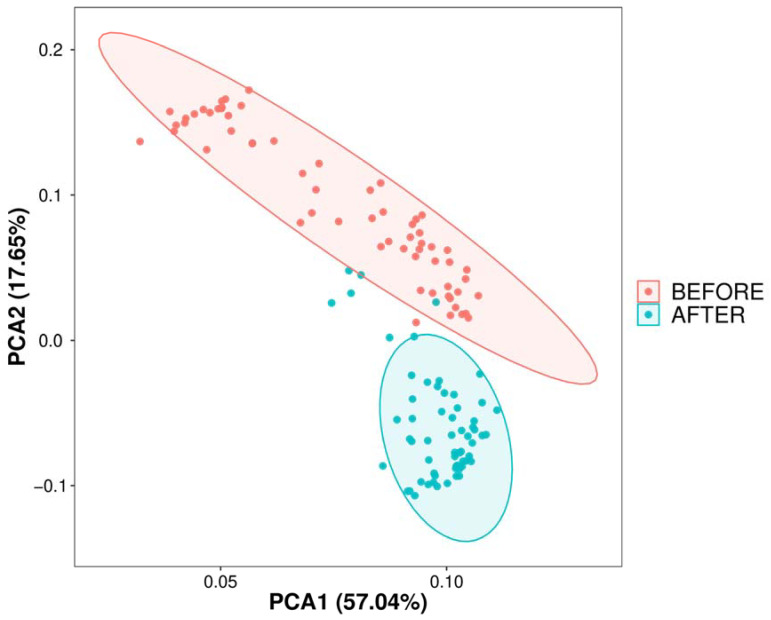
Dimensional reduction of species composition variability via PCA. PCA of sample differences based on ASV abundance: displays sample variability on a two-dimensional plot using PC1 and PC2, with species composition similarity dictating proximity on the plot; analysis performed with R’s vegan package.

Initially, we conducted a comparative analysis of the gut microbiota compositions pre- and post-anticancer therapy and employed amplicon sequence variants (ASVs) to monitor dynamic changes occurring in the bacterial abundances of different groups. In terms of alpha diversity, the violin plot (Figure 1) suggests that significant differences (*p* < 0.01) occurred in the overall gut microbiota abundance before and after anticancer therapy. Principal component analysis (Figure 2) also revealed significant differences in the beta diversity.

**Figure 3:**
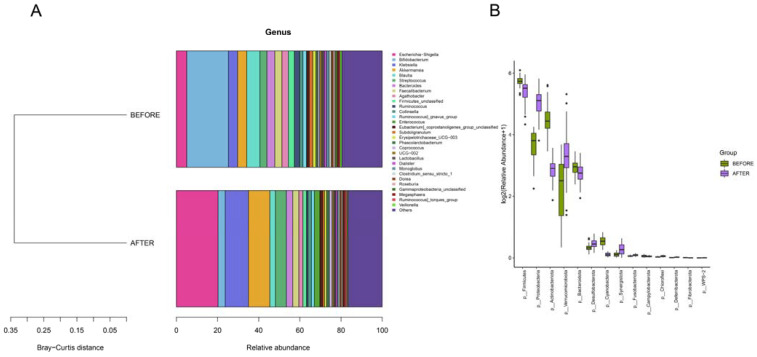
(**A**) “Bray-Curtis Clustering of Phylum-Level Species Composition”: illustrates phylogenetic proximity with shorter branches indicating higher similarity. (**B**) “Top 30 Phyla by Differential Abundance”: bar plots showing species ranked by relative abundance with significance indicated by *p*-values less than 0.05.

**Figure 4:**
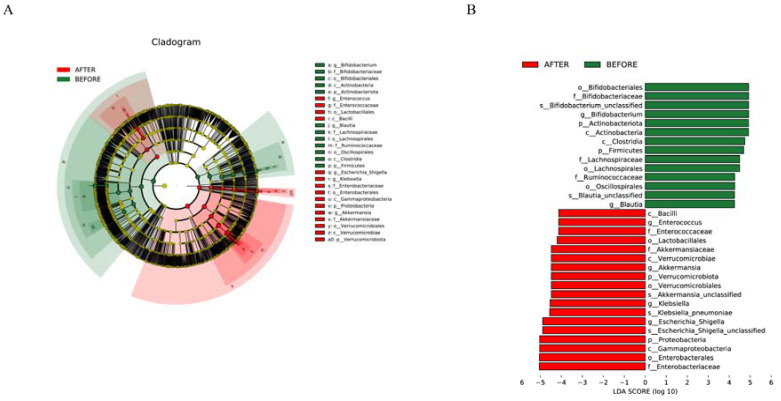
(**A**) “Multilevel Taxonomic Evolutionary Tree”: circular layers depict the hierarchy from kingdom to species; node size reflects species abundance, with color differences highlighting statistical significance in abundance across groups. (**B**) “Biomarker Distribution with LDA Scores”: bars illustrate biomarkers ex-ceeding an LDA threshold of 4.0, with color indicating higher abundance and length showing the biomarker’s influence.

We delved into the gut microbial community structure of both the pre- and post-treatment groups. Our analysis unveiled the dominance of phyla such as Firmicutes, Proteobacteria, Actinobacteriota, Verrucomicrobiota, and Bacteroidota. Notably, post-treatment observations revealed an escalated abundance of Proteobacteria and Verrucomicrobiota and decreased abundance of Firmicutes, Actinobacteriota, and Bacteroidota in comparison with that in the pre-treatment group. Significant differences in the abundances of Firmicutes, Proteobacteria, Verrucomicrobiota, and Bacteroidota were identified between the two groups. Furthermore, biomarker predictions showcased a decline in the abundances of Actinobacteriota and Firmicutes post-anticancer therapy, along with a surge in the abundance of Verrucomicrobiota (Figure 3 and Figure 4).

**Figure 5:**
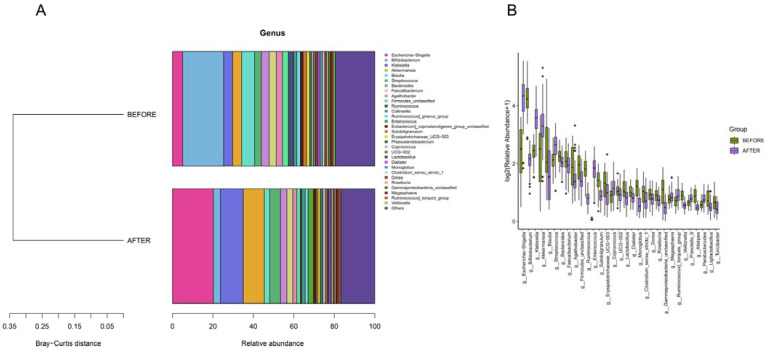
(**A**) “Genus-Level Bray-Curtis Clustering”: tree diagram showing sample clustering based on genus composition similarity, with shorter branches indicating closer relationships. (**B**) “Genus-Level Differential Abundance”: bar charts of the top 30 genera ranked by relative abundance, selected based on *p*-values below 0.05.

Genera such as Escherichia/Shigella, Bifidobacterium, Klebsiella, Akkermansia, Streptococcus, and others were consistent across both groups. The post-treatment group, however, demonstrated an uptick in the abundances of genera such as Escherichia/Shigella, Klebsiella, and Akkermansia, while showing a significant dip in the abundance of Bifidobacterium compared with that in its pre-treatment counterpart. Distinct differences in the abundances of several genera, including Escherichia/Shigella and Klebsiella, were discerned between the groups. Biomarker predictions at this level pinpointed a decrease in the abundances of Bifidobacterium and Blautia following anticancer therapy. Conversely, a rise was observed in the counts of Enterococcus, Akkermansia, Klebsiella, and Escherichia/Shigella, all of which have the potential to serve as NSCLC-anticancer therapy biomarkers. These bacterial species could be pivotal in preemptively addressing or treating complications that may surface during the therapeutic journey of NSCLC patients (Figure 4 and Figure 5).

### 3.3. Differentially Abundant Metabolites

**Figure 6:**
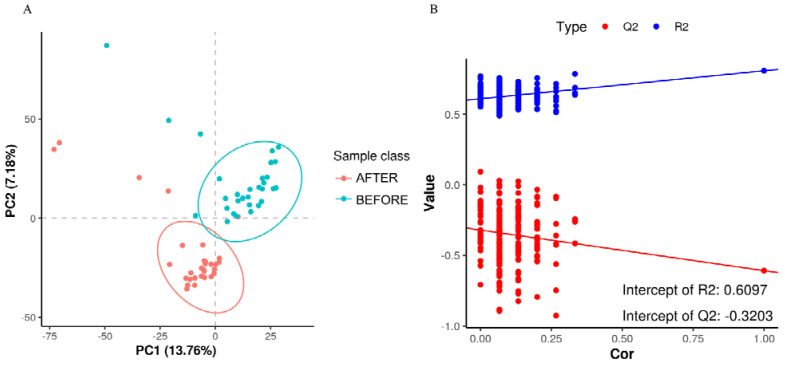
(**A**) “PLS-DA Score Plot”: illustrates sample classification along PC1 and PC2 with dispersion between groups indicating sample distribution trends. (**B**) “Permutation Test for Model Validation”: depicts the relationship between the original classification and permutation-derived classifications to assess model overfitting, with Q2 < 0 signifying no overfitting.

**Figure 7:**
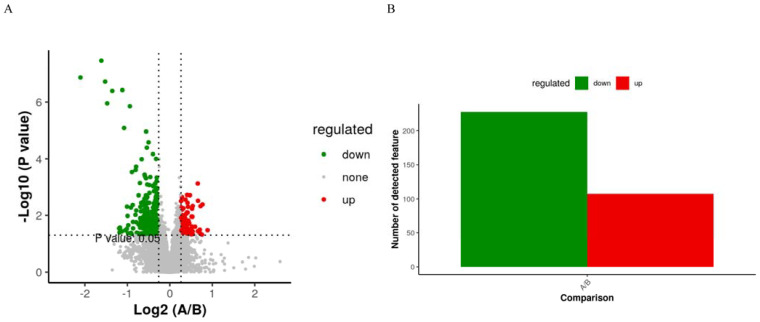
(**A**) “Volcano Plot of Differentially Expressed Metabolites”: showcases metabolites with significant fold changes and *p*-values, indicating up- or downregulation. (**B**) Summary of 107 upregulated and 228 downregulated metabolites, selected based on fold change, *p*-value, and PLS-DA VIP criteria.

The PLS-DA score plot showed a clear separation between the groups before and after treatment (Figure 6). The permutation test revealed no data overfitting, with the R2Y and Q2 values being 0.6097 and −0.3203, respectively, which validated the orthogonal PLS-DA model. The differentially expressed metabolic ions were screened using *t*-test *p*-values and variable importance in projection (VIP) values with thresholds of VIP ≥ 1.0 and *p* < 0.05. We detected 335 differential metabolites between the groups before and after treatment (Figure 7), most of which were downregulated after treatment.

**Figure 8:**
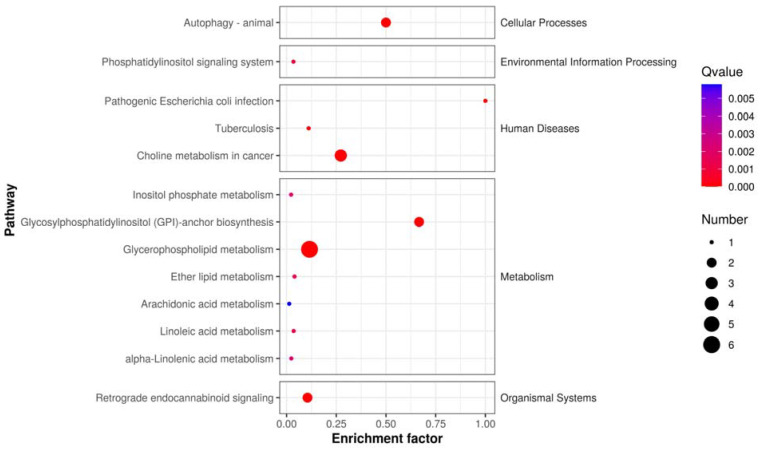
KEGG pathway enrichment scatter plot: depicts the rich factor of differentially expressed metabolites within specific pathways, with significance indicated by the *p*-value.

Metabolites with high differential fold changes primarily included lipids and lipid-like molecules, with glycerophospholipids and prenol lipids being downregulated after anticancer therapy compared with those before anticancer therapy and fatty acyls being upregulated (Figure 8). The results of Kyoto Encyclopedia of Genes and Genomes (KEGG) pathway enrichment analysis showed that the differentially abundant metabolites in patients with NSCLC before and after treatment were mainly enriched in the metabolism of inositol phosphate, glycerophospholipid, ether lipid, arachidonic acid, linoleic acid, alpha-linolenic acid; glycosylphosphatidylinositol (GPI)-anchor biosynthesis; and retrograde endocannabinoid signaling.

### 3.4. Correlation Analysis

**Figure 9:**
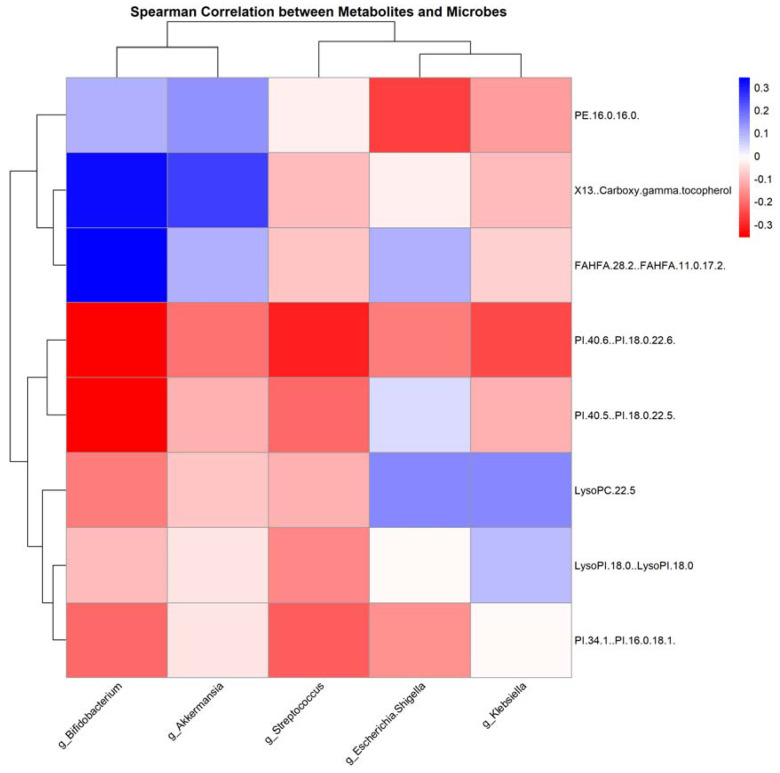
Correlation heatmap of gut microbiota and serum metabolites post-antitumor therapy: this heatmap illustrates the correlations between the levels of gut microbiota genera and serum metabolites following antitumor treatment, determined using Spearman’s correlation analysis; red indicates a positive correlation, while blue signifies a negative correlation.

**Figure 10:**
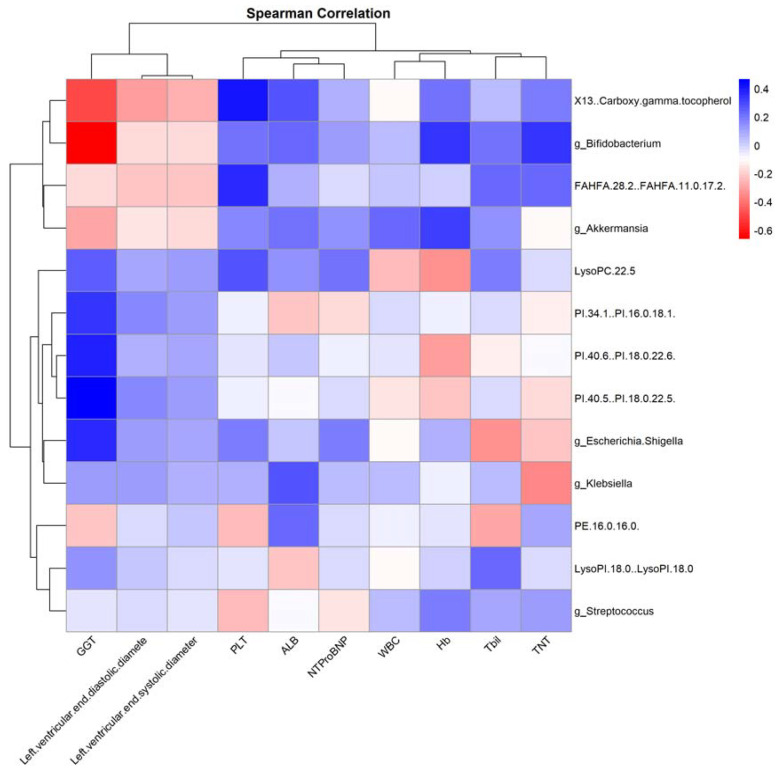
“Correlation Heatmap of Clinical Features with Gut Microbiota and Serum Metabolites Post-Antitumor Therapy”. This heatmap depicts the correlations between clinical features and both gut microbiota genera levels and serum metabolite levels after antitumor treatment, assessed using Spearman’s correlation analysis. Red indicates a positive correlation, while blue denotes a negative correlation.

To further investigate microbiota–metabolite interactions associated with anticancer therapy, we assessed correlations between five genera and eight metabolites. The abundance of several microbial genera was positively correlated with serum metabolite levels (Sperman’s correlation analysis, *p* < 0.05, Figure 9). Similarly, we analyzed the correlation between bacterial genera and clinical indicators with significant changes, and the results are represented as heat maps (Spearman’s correlation analysis, *p* < 0.05, Figure 10). 

### 3.5. Subgroup Analysis

Based on the initial treatment regimens of the patients, groups were established and a longitudinal comparison of the gut microbiome structure and serum metabolites was performed after four cycles. A mixed-effects model was employed for analysis, revealing no statistically significant differences across groups. Furthermore, based on the outcomes of the four-cycle anticancer treatment, patients were categorized into partial response (PR), stable disease (SD), and progressive disease (PD) groups. Longitudinal comparisons of the baseline microbiota and post-four-cycle treatment microbiota structures across these three groups also indicated no statistically significant differences.

In the subgroup analysis focusing on treatment regimens, including Platinum-based chemotherapy, Platinum-based chemotherapy combined with immune checkpoint inhibitors (ICIs), and Platinum-based chemotherapy combined with vascular endothelial growth factor inhibitors (VEGF inhibitors), alpha diversity metrics such as the Shannon index (*p* = 0.93) and Goods Coverage of microbial species (*p* = 0.85) showed no significant differences. Beta diversity, assessed through principal component analysis (PCA), also demonstrated no significant differences across these treatment subgroups. Further analysis at the phylum and genus levels revealed no significant differences in microbial abundance.

In the analysis of patient response categories—PR, SD, and PD—similar patterns were observed. The alpha diversity measures, including the Shannon index (*p* = 0.95) and Goods Coverage of microbial species (*p* = 0.91), did not show significant differences. Beta diversity, as evaluated by PCA, also did not reveal notable differences. Additionally, the analysis of microbial abundance at the phylum and genus levels did not identify any species with significant differences across these response groups.

## 4. Discussion

Lung cancer, specifically NSCLC, is the leading cause of cancer-related deaths globally. NSCLC treatment typically involves anticancer therapy, particularly cisplatin-based anticancer therapy and albumin-bound paclitaxel, which have proven efficacious. However, the effect of anticancer therapy on the gut microbiota and potential implications in patient health is an active research area [28].

The human gut microbiota, involving a complex ecosystem of trillions of microbes, has received considerable attention owing to its extensive influence on host physiology, nutrition, and immunity [29]. Increasing evidence suggests that gut microbiota composition can be significantly altered by anticancer therapy, potentially leading to various health consequences [30]. Post-cancer treatment, a noticeable alteration occurs in the fecal microbiota. In patients receiving chemotherapy, chemotherapeutic agents can damage the intestinal epithelial cells, leading to dysbiosis of the gut microbiome. This results in ectopic gut microbiota and severe disruption of the gut microbiota structure, characterized by a significant reduction in the abundance of anaerobic bacteria (such as Bacteroides, Clostridium, Prevotella, and Bifidobacterium) and Streptococcus. Concurrently, there is a marked increase in the population of Enterococcus [31].

Gut microbiota alterations during anticancer therapy have important health implications. Dysbiosis, an imbalance or maladaptation in the microbiota, is associated with anticancer therapy-induced gastrointestinal toxicity, which can lead to symptoms such as nausea, vomiting, and diarrhea [32]. These adverse effects can complicate treatment, lower the quality of life of patients, and potentially influence treatment outcomes [33]. The gut microbiota is also considered a potential biomarker for anticancer therapy-induced complications [34]. The role of the gut microbiota as a diagnostic or prognostic tool is an exciting research avenue, promising more personalized and effective patient management.

Based on the aforementioned existing research, we further investigated the changes in the gut microbiota of NSCLC patients after undergoing anticancer therapy. Firmicutes and Bifidobacterium (from phylum Actinobacteriota) are typically abundant in a healthy gut and are linked to positive health effects. Firmicutes contribute to the breakdown of complex carbohydrates and produce short-chain fatty acids beneficial for gut health [35]. Bifidobacterium is known for its role in preventing intestinal inflammation, enhancing barrier function, and producing vitamins [36]. Reduced abundance of Firmicutes and Bifidobacterium after anticancer therapy may affect these beneficial roles, potentially impacting gut health and overall patient wellbeing. However, the exact implications of these changes are complex and require further investigation.

Proteobacteria, which showed increased abundance after treatment, include pathogenic species such as Escherichia and Klebsiella [37]. These genera include opportunistic pathogens that can cause infections under immunocompromised conditions, such as those caused by anticancer therapy [38]. An increased abundance in Proteobacteria is typically associated with gut dysbiosis and has been linked to conditions such as inflammatory bowel disease and metabolic syndrome [39]. Thus, the increased abundance of Proteobacteria reflects a negative impact on the gut ecosystem due to anticancer therapy. 

The abundance of Akkermansia (belonging to Verrucomicrobiota) and Streptococcus also increased post-treatment. Akkermansia is generally considered beneficial and is associated with healthy gut function and anti-inflammatory properties [40]. However, the Streptococcus genus includes species that can be pathogenic or beneficial, complicating the interpretation of its increased abundance [41].

This study uncovered intriguing correlations between specific gut microbial genera and serum metabolites. Notably, there was a strong negative correlation between the genus Bifidobacterium and the metabolite PE.16.0.16.0, whereas Klebsiella exhibited a significant positive relationship with several metabolites, including LysoPC.22.5, PI.34.1..PI.16.0.18.1, and PE.16.0.16.0. These correlations are thought-provoking, especially in the context of NSCLC treatment regimens.

Bifidobacterium is widely recognized for its health-promoting effects in the human gut, mainly through the production of short-chain fatty acids (SCFAs) and modulation of the immune system [42]. An abundance of this genus has been linked to beneficial anti-inflammatory effects and improved gut barrier function [43]. In contrast, certain strains of Klebsiella are opportunistic pathogens that can lead to various infections and have been associated with inflammatory states [44].

PE.16.0.16.0, a phosphatidylethanolamine (PE), is involved in maintaining cell membrane integrity and fluidity [45]. A negative correlation between Bifidobacterium and PE.16.0.16.0 suggests that increased levels of this bacterium potentially enhance cellular health, particularly in the context of NSCLC, which is characterized by rapid cell turnover and cellular stress.

LysoPC.22.5 is a lysophosphatidylcholine involved in inflammation and cell signaling. Elevated levels of this metabolite have been associated with various pathologies, including cardiovascular diseases [46]. PI.34.1..PI.16.0.18.1 is a phosphatidylinositol, a lipid involved in cell signaling and membrane dynamics. Its positive correlation with Klebsiella indicates that an increased abundance of this genus may influence or be influenced by altered cellular signaling and inflammatory states [47].

In view of NSCLC treatments, platinum-based chemotherapy drugs are known to have myelosuppressive and immunosuppressive effects, potentially altering gut microbial composition [28]. Immune checkpoint inhibitors are designed to enhance the immune response against tumor cells, which might also interact with the gut microbiota’s immunomodulatory functions [11]. Anticancer therapy appears to significantly alter the gut microbiota in patients with NSCLC, potentially impacting gut health and overall wellbeing. Understanding these changes and their implications could help develop strategies to manage the side effects of anticancer therapy and improve patient outcomes.

Results also revealed that the white blood cell (WBC), hemoglobin (Hb), and platelet (PLT) levels were significantly lower in patients after anticancer therapy than in patients before anticancer therapy. These outcomes could be due to anticancer therapy-related myelosuppression. The levels of liver function-related indicators, particularly ALB, decreased significantly after anticancer therapy, which was likely related to the overall nutritional status of the patients. Significant differences in brain natriuretic peptide (BNP) and cardiac troponin T (cTnT) levels were also observed after anticancer therapy, which was potentially indicative of early myocardial damage.

An observation from our study is the positive correlation between Bifidobacterium and Hb. This finding is noteworthy, as several prior investigations have highlighted the health-promoting roles of Bifidobacterium, including its potential anti-inflammatory properties and contribution to gut homeostasis [48]. The enhanced Hb levels could hint at better overall health and reduced anemia, possibly indicating that patients with higher Bifidobacterium abundance might be in a better state. Furthermore, the metabolite FAHFA.28.2..FAHFA.11.0.17.2.’s strong negative relationship with cTnT underscores its potential role in mediating cardioprotective effects. Future studies should delve deeper into understanding its bioactivity and the mechanisms underlying this observed relationship. 

The results of our study provide important insights into the possible roles of the gut microbiota in anticancer therapy-related complications for patients with NSCLC. The dysbiosis of the gut microbiota induced by anticancer therapy may play a role in various adverse reactions. Previous research confirmed that the impact of anticancer therapy on the gut microbiota can lead to a series of pathological and physiological processes. For example, the results of a study of anticancer therapy-induced pain showed that the colonic microbiota promoted the development of anticancer therapy-induced mechanical hypersensitivity [49]. Another study reported that some bacterial species were depleted after anticancer therapy, which may lead to psychosocial and cognitive problems [30]. Understanding the biological mechanisms underlying psychosocial and cognitive dysfunctions in patients caused by gut microbiota dysbiosis could help in developing targeted interventions. 

Huang et al. [34] used a mouse model to demonstrate that glycyrrhizic acid (GLA) reduced the leakage of myocardial enzymes (including transaminases, creatine kinase, lactate dehydrogenase, and creatine kinase-MB) caused by doxorubicin. An et al. [50] found that mice that received a gut microbiota transplantation showed improved cardiac function, reduced intestinal injury, and a restored microbiota balance during doxorubicin treatment. The gut microbiota transplantation mitigated the side effects of doxorubicin on the heart and intestine and provided a therapeutic strategy for preventing anticancer therapy-induced heart failure and intestinal injury [50]. However, further research is necessary to elucidate the causal relationship between anticancer therapy and gut microbiota alterations. 

The current study has a few shortcomings, mainly owing to the limitations in sample size and follow-up time. Our research on various complications remains at the examination level, indicating a necessity for further follow-up and discussion on major anticancer therapy-related complications. Furthermore, the follow-up points in this study for the intestinal microbiota appear somewhat sparse. More points might allow for the analysis of changing trends among different microbial communities. Large sample sizes should be studied to continue exploring the related metabolomics and search for possible biomarkers. It will also be important to design more rigorous randomized controlled trials targeting different anticancer therapy-related complications, such as myocardial injury, to explore the potential of microbial taxa as therapeutic targets and biomarkers of treatment responses. 

Previous data showed that gut microbiota dysbiosis promoted the onset of gastrointestinal malignancies through different pathways, that is, regulating human metabolism and participating in immune processes and inflammatory reactions [51]. In particular, alterations in the gut microbiota composition have been observed in patients with gastrointestinal tumors before the onset of disease. A case-control study on colon cancer involving 131 individuals showed a positive correlation between a significantly decreased abundance of Lactobacillaceae and increased abundance of Clostridium and Porphyromonas and the levels of palmitoyl phosphatidylcholine and 4-hydroxybenzaldehyde [52]. Therefore, bidirectional interactions exist between the gut microbiota and gastrointestinal tumors, indicating that future research should be conducted to investigate complications related to tumor treatment involving the gut microbiota and account for the influence of gastrointestinal tumors.

As we did not observe significant differences in bacterial species and metabolites among the various subgroups delineated by treatment plan and therapeutic efficacy, this may be attributed to the limitations in sample size or insufficient follow-up duration. Consequently, it was not feasible to conduct a more in-depth correlation analysis among the bacterial flora, metabolites, and clinical characteristics. This indicates that future studies should consider expanding the sample size and further optimizing the inclusion criteria to enable a more comprehensive investigation and exploration of the nuances related to different treatment regimens and their therapeutic outcomes. Additionally, the treatment regimens of our study subjects were based on chemotherapy drugs. Future research could explore the impact of single-agent therapies such as immune checkpoint inhibitors on the gut microbiome and metabolic products.

## 5. Conclusions

In conclusion, the results of our study highlight the significance of gut microbiota changes in NSCLC patients undergoing anticancer therapy. The alterations in abundance of Escherichia/Shigella, Klebsiella, Akkermansia, Streptococcus, and Bifidobacterium observed in our patient cohort have potential implications for preventing and managing anticancer therapy-related complications. Future research should focus on establishing causal relationships between anticancer drug therapy and gut microbiota alterations and evaluating potential strategies to counteract these changes and improve patient outcomes. The sample size and scope of the study should be expanded, including research on larger cohorts, comparative analyses of different types of cancer, and more detailed and isolated analysis of patient groups receiving varying treatment modalities.

## Figures and Tables

**Table 1:** Inclusion and exclusion criteria.

Inclusion Criteria	Exclusion Criteria
Diagnosed with non-small cell lung cancer	Has a gastrointestinal disease such as inflammatory bowel disease or a gastrointestinal tumor
Will receive anticancer drugs	Also has a cardiovascular disease such as coronary heart disease, valvular disease, or arrhythmia
Can undergo regular follow-ups for 4 cycles	Also has acute kidney injury or chronic kidney disease
Written informed consent provided	Also has hepatic insufficiency
	Participating in another drug intervention study

**Table 2:** General clinical data.

Parameter	
Age, years, median (Q1–Q3)	65 (45–75)
Male, *N* (%)	46 (76.7)
Pathological type	
Squamous cell carcinoma, *N* (%)	34 (56.7)
Adenocarcinoma, *N* (%)	25 (41.7)
Large cell carcinoma, *N* (%)	1 (1.6)
Stage	
Stage III, *N* (%)	28 (46.7)
Stage IV, *N* (%)	32 (53.3)
Treatment regimen	
Platinum-based chemotherapy, *N* (%)	26 (43.3)
Platinum-based chemotherapy combined with immune checkpoint inhibitors, *N* (%)	25 (41.7)
Platinum-based chemotherapy combined with vascular endothelial growth factor inhibitors (VEGF inhibitors), *N* (%)	9 (15.0)
Taxane-based chemotherapy, *N* (%)	38 (63.3)
Therapeutic effect	
Partial response (PR), *N* (%)	44 (73.3)
Stable disease (SD), *N* (%)	11 (18.3)
Progressive disease (PD), *N* (%)	5 (8.4)

**Table 3:** Clinical parameters.

**Parameter**	Before	After	*p*-value †
**Complete blood count**			
WBC (×10^9^/L)	7.09 ± 2.46	5.98 ± 2.26	0.002 **
Hb (g/L)	125.31 ± 22.69	114.81 ± 13.83	0.003 **
PLT (×10^9^/L)	233.94 ± 95.52	207.05 ± 53.32	0.042 *
**Liver function**			
ALT (U/L)	62.34 ± 31.96	63.18 ± 31.18	0.863
AST (U/L)	80.55 ± 33.29	80.74 ± 32.18	0.975
TBIL (μmol/L)	9.91 ± 4.65	11.93 ± 5.71	0.040 *
ALB (g/L)	41.84 ± 7.73	38.48 ± 6.25	0.012 *
GGT (U/L)	29.76 ± 12.1	35.50 ± 15.75	0.018 *
ALP (U/L)	58.26 ± 28.48	60.35 ± 25.40	0.677
**Renal function**			
BUN (mmol/L)	4.92 ± 1.62	5.09 ± 1.51	0.595
Cr (mmol/L)	76.74 ± 21.93	73.95 ± 21.15	0.498
**Markers of myocardial injury**			
NT-proBNP (pg/mL)	225.48 ± 97.33	275.11 ± 115.623	0.015 *
cTnT (pg/mL)	0.008 ± 0.039	0.011 ± 0.006	0.011 *
CK-MB (U/L)	17.69 ± 4.45	19.23 ± 4.54	0.094
**Echocardiography**			
Left atrial antero-posterior diameter (mm)	33.29 ± 6.15	33.75 ± 6.30	0.530
Left atrial area (cm^2^)	18.20 ± 5.69	19.02 ± 5.81	0.183
Right atrial area (cm^2^)	13.87 ± 2.50	14.27 ± 2.49	0.147
Interventricular septal thickness (mm)	7.55 ± 1.28	7.62 ± 1.20	0.808
Left ventricular posterior wall thickness (mm)	7.74 ± 0.53	7.79 ± 0.55	0.455
Left ventricular end-diastolic diameter (mm)	48.83 ± 6.51	50.26 ± 6.32	0.040 *
Left ventricular end-systolic diameter (mm)	28.94 ± 4.62	30.11 ± 4.61	0.017 *
Right ventricular antero-posterior diameter (mm)	20.40 ± 2.25	20.62 ± 2.22	0.406
Left atrial pressure (mmHg)	7.23 ± 2.49	7.84 ± 2.81	0.039 *
LVEF (%)	70.87 ± 4.25	70.63 ± 4.07	0.583

ALB—albumin; ALP—alkaline phosphatase; ALT—alanine aminotransferase; AST—aspartate aminotransferase; BUN—blood urea nitrogen; CK-MB—creatine kinase isoenzyme MB; Cr—creatinine; cTnT—cardiac troponin T; GGT—γ-glutamyl transaminase; Hb—hemoglobin; NT-proBNP—N-terminal brain natriuretic peptide precursor; PLT—platelet; TBIL—total bilirubin; WBC—white blood cell. † * *p* < 0.05, ** *p* < 0.01

## Data Availability

The data generated in this study are available upon request from the authors.
